# Rapid Impregnating Resins for Fiber-Reinforced Composites Used in the Automobile Industry

**DOI:** 10.3390/polym15204192

**Published:** 2023-10-23

**Authors:** Mei-Xian Li, Hui-Lin Mo, Sung-Kwon Lee, Yu Ren, Wei Zhang, Sung-Woong Choi

**Affiliations:** 1 School of Textile and Clothing, Nantong University, Nantong 226019, China; lmx321@ntu.edu.cn (M.-X.L.);; 2 National and Local Joint Engineering Research Center of Technical Fiber Composites for Safety and Protection, Nantong University, Nantong 226019, China; 3 Department of Mechanical System Engineering, Gyeongsang National University, Tongyeong-si 53064, Gyeongsangnam-do, Republic of Korea

**Keywords:** rapid impregnation, thermoset resins, thermoplastic resins, fiber-reinforced composites

## Abstract

As environmental regulations become stricter, weight- and cost-effective fiber-reinforced polymer composites are being considered as alternative materials in the automobile industry. Rapidly impregnating resin into the reinforcing fibers is critical during liquid composite molding, and the optimization of resin impregnation is related to the cycle time and quality of the products. In this review, various resins capable of rapid impregnation, including thermoset and thermoplastic resins, are discussed for manufacturing fiber-reinforced composites used in the automobile industry, along with their advantages and disadvantages. Finally, vital factors and perspectives for developing rapidly impregnated resin-based fiber-reinforced composites for automobile applications are discussed.

## 1. Introduction

Recently, lightweight automobiles have been increasingly used to save energy and reduce pollution in light of strict regulations. It has been reported that reducing automobile weight by 10 wt.% could decrease fuel consumption by 6–8% and reduce CO_2_ emissions [1]. The key to solving this problem is to replace metal components with high-performance, fiber-reinforced, polymer-based composites. As for environmental issues, extensive research has been conducted on biocompatible and environmentally-friendly resins, including the use of the poly (ethylene glycol) diacrylate (PEGDA) monomer [2], their applications in dental materials [3], and their utilization in 3D printed materials [4,5,6]. This research focuses on polymer composite molding processes, and understanding these molding processes is essential. 

Polymer composites, including thermosets and thermoplastic composites, have been used in panels, modules, structures, and other parts of automobiles after being reinforced with continuous or discontinuous fibers that have undergone liquid composite molding (LCM) processes, such as resin transfer molding (RTM), vacuum infusion, and reaction injection molding [7]. Takahashi et al. [8] reported that the weight of carbon fiber reinforced polymer (CFRP) composites is one third that of steel panels, and the flexural strength of CFRPs is approximately three times higher. As for the polymer composite, fiber-reinforced thermoset composites (FRTSCs) generally exhibit good mechanical properties, thermal stability, and dimensional stability [9]. Thermoset resins have a relatively low viscosity compared with thermoplastic resins, which is an important factor in the RTM process. Traditionally, depending on the part size and geometry, the cycle time of a standard RTM is 30–60 min with a 10–20 bar injection pressure, whereas that of high-pressure resin transfer molding is less than 10 min with a 20–120 bar injection pressure.

Fiber-reinforced thermoplastic composites (FRTPCs) are also extensively used because of their high processability and recyclability. However, the high viscosity of thermoplastic resins requires high temperatures and pressures for the materials to be impregnated with fiber reinforcements. For example, thermosetting resins, such as epoxy, can be impregnated with fiber reinforcements and cured below 200 °C, whereas thermoplastic resins must be heated above the melting temperature, which is typically well above 200 °C, and impregnated with high pressures (10–50 bar). Moreover, the high pressure and viscosity of the resins may cause a misalignment of the fiber reinforcements.

In the automobile industry, cost and cycle time reductions are key issues. In the case of thermoset resins, there is a need for research into fast-curing resins like rapid curing epoxies and endo-dicyclopentadiene. On the other hand, for thermoplastic resins, research is required on the development of rapidly impregnating resins that can be applied at low temperatures and low pressures. Regarding fast-curing thermoset resins, recent research by Zhang et al. [10], Odom et al. [11], Gan et al. [12], and Reichanadter et al. [13] presented the application of fast-curing epoxy resins and its processes. Boros, Róbert, et al. [14], Ota et al. [15], and Willicombe, K., et al. [16] showed the rapid impregnation process and feasibility of a new approach using thermoplastic resin. In many instances, research tends to emphasize methodologies and approaches that are focused on specific manufacturing systems. Consequently, it can be challenging to find examples and studies that provide a comprehensive exploration of various types of resins. 

The main objective of the present study is to provide a comprehensive overview of various types of fast-curing and rapidly impregnating resins using a broad array of resin cases. In the present study, a comprehensive overview of commonly used, rapidly impregnating, low-viscosity resins in the automobile industry was provided, including thermosets and thermoplastic resins. Additionally, various reactive processes, and the parameters that influence them, were presented, alongside an examination of the properties associated with Fast-Curing Resin Thermosetting Composites (FRTSCs) and Fast-Curing Resin Thermoplastic Composites (FRTPCs). Finally, the current status of related research and insights into future perspectives in this field were addressed.

## 2. Rapidly Impregnating Resins and Their Fiber-Reinforced Composites

### 2.1. Knowledge Gaps and Current Challenges

Numerous methods for rapidly impregnating thermoset and thermoplastic resins in fiber-reinforced composites have focused their attention on various aspects requiring further development. This section presents the main knowledge gaps that need to be filled in order to address the current challenges regarding the rapid impregnation of thermoset and thermoplastic resins and their fiber-reinforced composites, to understand the development method and the its applications.

Thermoset resins, such as epoxy, polyester, and vinyl ester, are used in a wide range of automobile parts, such as headlamp housings, battery covers, and frames for windows or sunroofs. Thermoset composites have excellent dimensional and chemical stabilities and high impact strengths, which are necessary for the interior and exterior parts of automobiles. In this section, thermoset resins and their fiber-reinforced composites are discussed.

**Figure 1:**
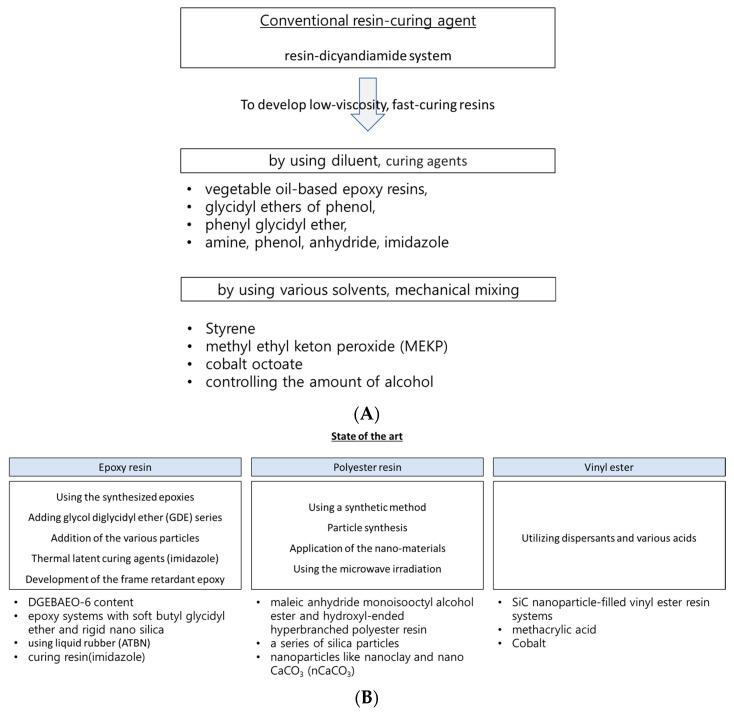
Knowledge Gap: (**A**) Conventional resin-curing system [18,19,20,21,22,23,24,25,26,27,28]. (**B**) State-of-the-art thermoset resin [25,26,27,28,29,30,31,32,33,34,35,36,37].

If the manufacturing cycle time can be reduced by using thermoset resin, several advantages become particularly noteworthy. To address these issues, various state-of-the-art methods have been introduced to achieve the fast impregnation of thermoset resin, as shown in Figure 1. The current state-of-the-art method for the rapid impregnation of thermoset resins and their fiber-reinforced composites primarily involves material selection, including the choice of resin and curing agent, as well as the utilization of advanced mixing techniques. Utilizing optimal materials (such as the epoxy resin with phenolic novolac epoxy and bisphenol-A epoxy, and the curing agent aliphatic polyamine dicyandiamide (DICY)), a specific manufacturing process was applied to achieve the rapid impregnation of the resin into the reinforcing fibers for resin transfer molding (RTM), vacuum infusion, and the reaction injection molding process [7]. The approach to achieving a fast and cost-effective impregnation and curing process was closely related to factors such as curing time, gel time for the resin, and curing kinetics. Consequently, finding most suitable resin and curing agent ultimately resulted in a substantial reduction in manufacturing costs (10~20% reduction in fiber cost, mandrel cost, tooling cost, system set up cost/process time under 240 min.) [17].

The current state-of-the-art rapidly impregnating thermoplastic resins and their fiber-reinforced composites have been tested with impregnation techniques, material selections, and processing parameters, etc. Several impregnation techniques, such as melt impregnation, powder impregnation, and resin transfer molding, have been explored to achieve the rapid impregnation of thermoplastic resins into fiber reinforcements; they have shown promise in terms of achieving efficient impregnation, reducing cycle times, and enhancing the overall quality of the composite [38,39]. Generally, higher temperatures can lower the viscosity of thermoplastic resins and pressure also helps to drive the resin into the fiber reinforcement, thus enabling better fiber impregnation and complete wetting, as well as the removal of void trapped within the fiber reinforced composites. However, excessively high temperatures may lead to resin degradation, and excessive pressure may lead to fiber deformation or damage. Therefore, optimizing the processing parameters is essential for achieving uniform resin distribution, complete fiber wetting, and strong interfacial bonding between the matrix and reinforcement, leading to the minimization of void content, as well as enhanced mechanical properties [40,41].

### 2.2. Thermoset Resins

#### 2.2.1. Epoxy

Epoxy resins have been used in the automobile industry since the 1980s, owing to their superior mechanical properties, low shrinkage and creep, and outstanding chemical resistance. The estimated size of the global epoxy resin market was USD 12.5 billion in 2021, and it is anticipated to reach approximately USD 23.4 billion by 2030, with an expected annual growth rate (CAGR) of 7.22% during the forecast period from 2022 to 2030 [18]. A representative commercial epoxy resin is the epoxy-dicyandiamide system, and many strategies have been implemented to develop low-viscosity, fast-curing epoxy resins. 

Conventionally, low viscosity can be achieved and controlled by incorporating various diluents, such as epoxy-based reactive diluents, which participate in the polymerization reaction and contribute to the cross-linking network. For example, the preferred viscosity range for the resins used in manufacturing composite materials via liquid molding is generally between 200 and 1000 cP at room temperature for 2~3 h of curing time [42]. Epoxy-based reactive diluents come in various forms, including vegetable oil-based epoxy resins, glycidyl ethers of phenol and paraalkyl substituted phenols, vinylcyclohexane dioxide, the phenyl glycidyl ether, and the trimethylol propane triglycidyl ether [19,20,21,22,23,24]. In addition, by introducing the catalytic mechanisms wherein epoxy crosslinks with the curing agent, a rapid curing time below 3 h can be obtained with tertiary amines.

**Figure 2:**
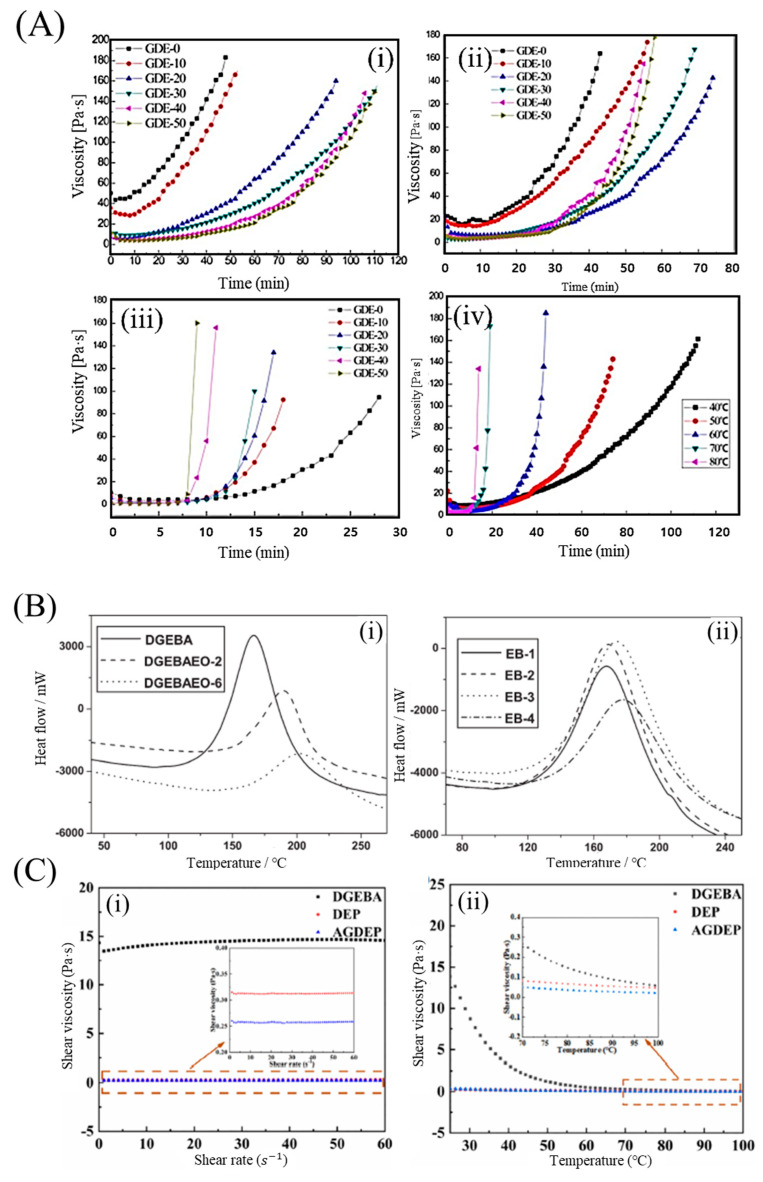
(**A**) Effect of glycol diglycidyl ether (GDE) content on the rheological behavior of the acrylate−based epoxy resin (AE)−40/LPA system: (**i**) 40 °C, (**ii**) 50 °C, (**iii**) 80 °C, (**iv**) 20 phr GDE content. Reproduced with permission [43]. Copyright 2015, John Wiley and Sons. (**B**) DSC curves for (**i**) three neat epoxy resins and (**ii**) DGEBA/DGEBAEO−6 cured using DDM. Reproduced with permission [26]. Copyright 2011, John Wiley and Sons. (**C**) Rheological behaviors of epoxies (without curing agent): (**i**) Change in viscosity, with shear rate from 0.01 to 60 s^−1^ at 25 °C, (**ii**) Change in viscosity with temperature from 25 to 100 °C at a shear rate of 60 s^−1^. Reproduced with permission [47]. Copyright 2022, John Wiley and Sons.

Fast-curing epoxy resins can be obtained by adding glycol diglycidyl ether (GDE) series. For example, a low-viscosity acrylate-based epoxy resin (AE)/GDE system was developed by Yang et al., and its rheological behavior is shown in Figure 2A [43]. Seraji et al. [25,44,45] developed a rapid-curing epoxy amine resin with low viscosity, which consists of the diglycidyl ether of bisphenol F, an epoxy phenolic novolac resin, diethyl toluene diamine, and 2-ethyl-4-methylimidazole. The resin system exhibited good thermal and mechanical properties, and superior flame retardancy. Based on these trends, low-viscosity, fast-curing epoxy resins were obtained using the synthesized epoxies. Two resins, the diglycidyl ether of ethoxylated bisphenol-A (BPA) with two and six oxyethylene units (DGEBAEO-2 and DGEBAEO-6), respectively, were synthesized and characterized; the curing exothermic enthalpy decreased with increasing oxyethylene units (Figure 2B) [26]. The viscosities of the blends decreased as the DGEBAEO-6 content increased. In addition, difunctional aromatic epoxy-divinylbenzene dioxide, which was synthesized with epoxidizing divinylbenzene as the catalyst, had a low molecular weight and viscosity, as well as excellent thermal (T_g_ was approximately 201 °C) and mechanical properties (tensile strength was 131.99 MPa). Wu, Xiankun, et al. [27] and Chen et al. [29] developed a series of epoxy systems with a soft butyl glycidyl ether and rigid nano silica, and a viscosity lower than 600 mPa·s, thus providing an excellent processing performance for the large-scale production of composites in automobile manufacturing. In addition, this system demonstrated improvements in terms of tensile strength and modulus, as well as in elongation at break. Wang et al. [46] reported an epoxy resin-1-(cyanoethyl)-2-ethyl-4-methylimidazol system. The epoxy cured in a few minutes at 120 °C with an acceptable pot life and low water absorption.

The reaction time decreased with the addition of the various particles. Chikhi et al. [30] developed a modified epoxy resin using liquid rubber (ATBN). All reactivity characteristics (gel time, temperature, curing time, and exothermic peaks) decreased. The addition of ATBN led to a reduction in either the glass transition temperature or the stress at break, accompanied by an increase in the elongation at break and the appearance of yielding. Zhang et al. [48] designed a tetrafunctional eugenol-based epoxy resin with a cyclosiloxane structure. Allyl glycidyl ether was selected as the reference compound to generate a silylation epoxy resin. The viscosity of the silicone-containing tetrafunctional epoxy monomers (<0.315 Pa·s) was significantly lower than that of conventional oil-based epoxy resins (14.320 Pa·s) (Figure 2C) [43].

Moreover, the low viscosity of epoxy resin-based component epoxy systems has recently been obtained for thermal latent curing agents and flame-retardant epoxies (generally below 200 cP at room temperature) [25,26,27,42]. Thermal latent curing agents of Imidazole are widely employed to fabricate single-component epoxy systems, and they meet the requirements for large-scale industrial production [49,50,51]. Several phosphorus-modified imidazole derivatives have been developed to combine fast curing rates (below 3 h [25,26,27] and great flame retardancy characteristics [52,53,54].

#### 2.2.2. Polyester

Regarding polyester resins, low-viscosity polyester resins can be obtained via particle synthesis. Low-viscosity polyester resins can be applied to produce environmentally friendly coatings, as well as to toughen and reinforce unsaturated polymers. The global market size of unsaturated polyester resins was estimated to be USD 12.2 billion in 2022, and it is expected to grow at an annual growth rate (CAGR) of 7.1% from 2023 to 2030 [55]. 

Traditionally, low-viscosity polyester resins can be obtained through various methods, including the use of solvents, mechanical mixing methods, etc. For instance, solvents such as styrene, methyl ethyl ketone peroxide (MEKP), and cobalt octoate are typically used. Control and mixing methods involving alcohol are frequently utilized. Nurazzi et al. [56] developed a method to reduce the gel time of unsaturated polyester (UPE) by blending it with methyl ethyl ketone peroxide (MEKP) and various percentages of cobalt. Using this method, the gel time can be reduced by up to 36%.

Recently, alternative approaches have been employed to achieve a lower viscosity (<300 mPa·s) in the compound [57], which facilitates the formation of crosslinking networks. These methods include synthetic techniques, particle synthesis using nanomaterials, microwave irradiation, among others [58,59].

**Figure 3:**
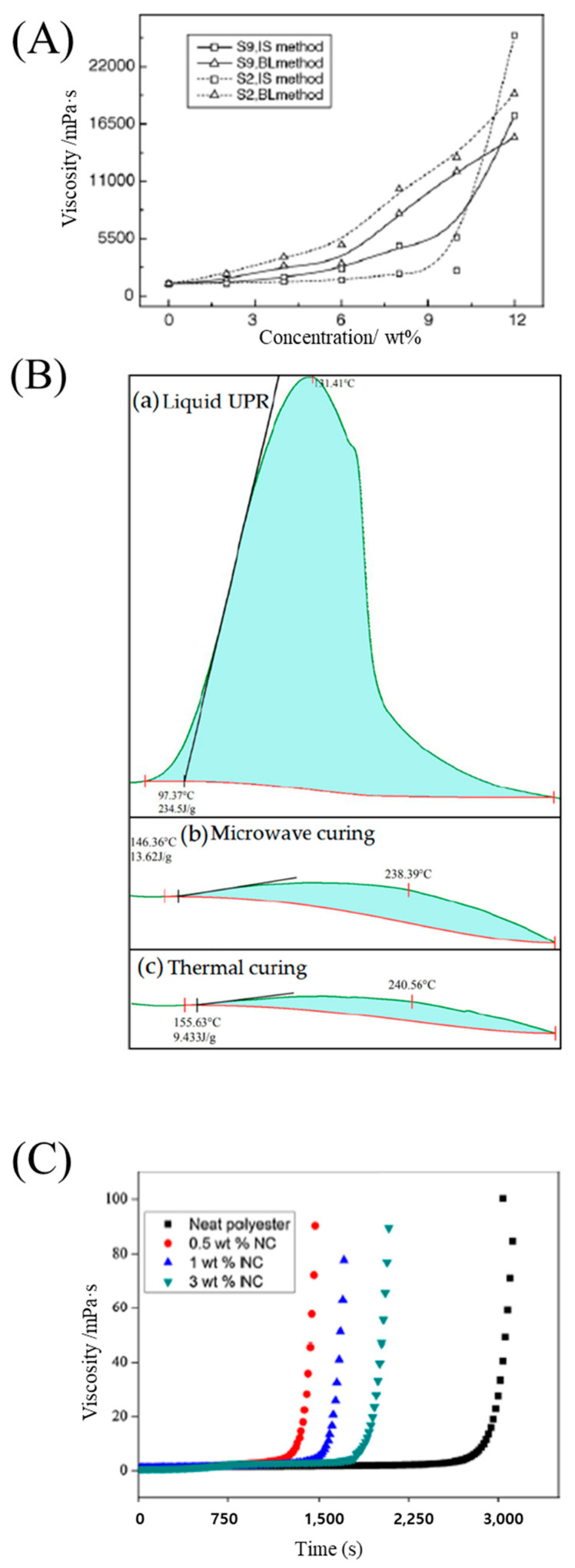
(**A**) Effect of silica content on the viscosity of nanocomposite resins embedded with silica sol S2 or S9. Reproduced with permission [31]. Copyright 2005, Elsevier. (**B**) DSC curves of liquid UPR, and cured samples with microwave curing and thermal curing, respectively. Reproduced with permission [32]. Copyright 2022, MDPI. (**C**) Variation of viscosity over time for NC filled composites. Reproduced with permission [61]. Copyright 2014, Elsevier.

Chen et al. [31] prepared a series of silica particles with different sizes and surface groups through the sol–gel process, using tetraethyl orthosilicate, and they were directly introduced into polyester polyol resins via in situ polymerization. The resulting nanocomposites exhibited lower viscosities than the resins obtained using the blending method. Viscosity increased as the particle concentration increased (Figure 3A). Zhang et al. [28] examined a low-viscosity unsaturated hyperbranched polyester resin (<10,000 cP) using a synthetic method involving a reaction between a maleic anhydride monoisooctyl alcohol ester and a hydroxyl-ended hyperbranched polyester resin prepared from phthalic anhydride and trimethylolpropane. Zhou et al. [57] synthesized a series of unsaturated polyester resins with low viscosities (<300 mPa·s), for a vacuum infusion molding process, by simply controlling the amount of alcohol used in the reactants. Yuan et al. [60] developed a series of low-viscosity transparent UV-curable polyester methacrylate resins, derived from renewable biologically fermented lactic acid (LA), and they reduced the viscosity from 34,620 mPa·s to 160–756 mPa·s by randomly copolymerizing LA and-caprolactone.

The curing time can be reduced using various solvents and applying microwaves. Nasr and Abdel-Azim [33] investigated unsaturated polyester resins, and styrene, methyl ethyl keton peroxide (MEKP), and cobalt octoate were selected as the solvent (monomer), catalyst, and accelerator, respectively. A significant reduction in curing time occurred when the cobalt octoate concentration was increased to 0.02 wt.%. Furthermore, the curing time decreased when the catalyst concentration was increased from zero to 2 wt.%. Mo et al. [32] applied microwave irradiation to the curing of an unsaturated polyester resin with CaCO_3_ particles, and they showed that microwave irradiation heated the unsaturated polyester resin evenly and rapidly, causing a chain growth reaction which greatly reduced the curing time (Figure 3B). Chirayil et al. [61] prepared nanocellulose-reinforced unsaturated polyester composites via mechanical mixing. The curing time required for gelation in the nanocellulose-filled unsaturated polyester was lower than that for the neat resin, indicating the catalytic action of nanocellulose in the curing reaction (Figure 3C). Kalaee et al. [34] utilized the nanoparticle of CaCO_3_ (nCaCO_3_) and found that a decrease in the number of carboxyl groups in the formulation leads to a higher degree of crosslinking.

#### 2.2.3. Vinyl Ester

The extensive use of vinyl ester resins as matrix materials in reinforced composites is due to their low viscosity, rapid curing capabilities at room temperature, and cost-effective advantages. Typically, in highly viscous vinyl ester resins, a low-viscosity environment can be achieved by utilizing dispersants and various acids, which effectively reduce their surface activities.

**Figure 4:**
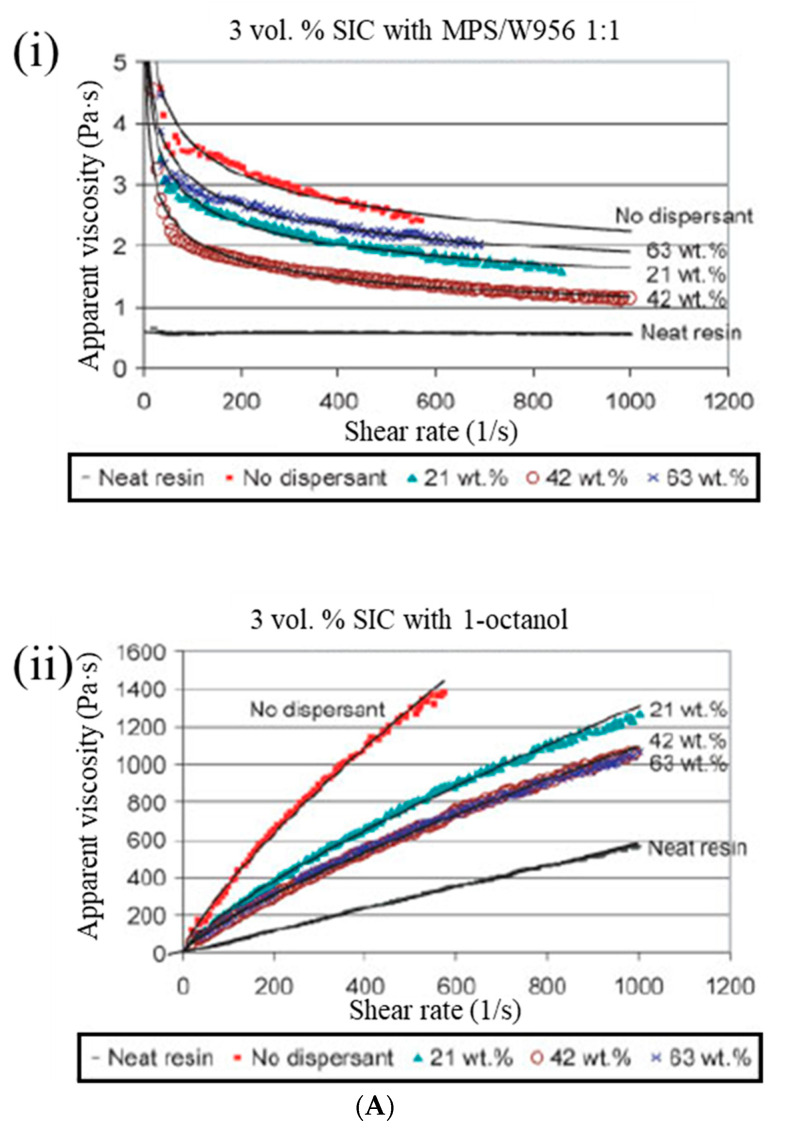

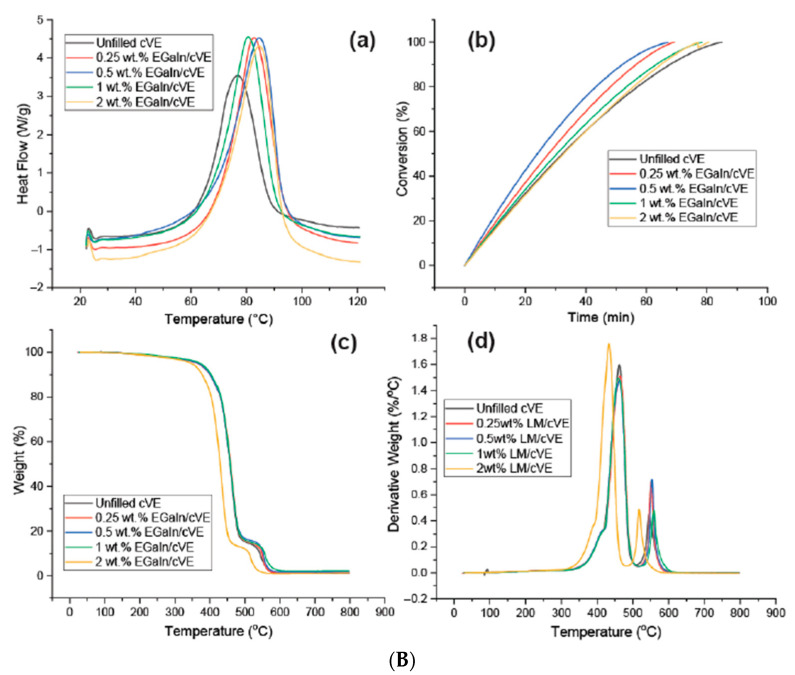
(**A**) (**i**) Viscosity curves of SiC/vinyl ester resin systems with and without MPS/W966. (**ii**) Viscosity curves of SiC/vinyl ester resin systems with and without 1-octanol. Reproduced with permission [35]. Copyright 2006, John Wiley and Sons. (**B**) The DSC graphs (**a**), degree of conversion at 60 °C (**b**), graphs of TGA (**c**), and DTG (**d**) at a heating rate of 5 °C/min for the LM filled and unfilled comonomer vinyl ester composites. Reproduced with permission [62]. Copyright 2022, MDPI.

Yong and Hahn [35] conducted a rheological analysis of SiC nanoparticle-filled vinyl ester resin systems using the Bingham, power law, Herschel–Bulkley, and Casson models. The incompatibility between a hydrophilic SiC and a hydrophobic vinyl ester resin can act as the driving force for the formation of SiC aggregates, even when low particle loading occurs (<0.04 volume fraction), resulting in the high viscosity of the resin. The optimum fractional weight percentage of dispersants (wt.% dispersant/wt.% SiC) for dispersion stabilization is 1–3% for particles in the 0.1–3-μm range, and it can be proposed, as follows: the addition of a dispersant at the optimum dosage lowers the viscosity of SiC/vinyl ester suspensions by 50% (Figure 4A).

Gaur et al. [36] obtained the zero-shear viscosity of vinyl ester resins containing styrene (40 wt.%) as the reactive diluent. The curing of vinyl ester resins can be controlled by reacting the epoxy novolac resin with methacrylic acid. They found that the curing and decomposition behavior of vinyl ester resins worsened with an increase in methacrylic acid content (11, 22, 32, 38, and 48 mg KOHg^−1^ solid). The cured product with the lowest acid value was the most thermally stable product. Cook et al. [37] analyzed the gel time and reaction rate of a vinyl ester resin and found that the cobalt species played a dual role in initializing the formation of radicals from MEKP and destroying primary and polymeric radicals. Based on these results, the reaction rate (determined using differential scanning calorimetry, (DSC)) increased and the gel time decreased with increasing concentrations of MEKP. However, cobalt octoate cocatalyst slows the reaction rate, except at very low concentrations. The gel time decreased as MEKP and cobalt octoate concentrations increased. Curing vinyl ester resins with modified silicone-based additives was achieved by Mazali et al. [63]. Silicone-based additives were used to modify the properties of the vinyl ester resin. For the resin cured in the absence of N, N-dimethylaniline, the silicone-based additives acted as retardants of the curing reaction, which is a typical diluent effect, whereas in the presence of this promoter, the reaction enthalpy and rate improved.

The viscosity of the vinyl ester resin could be reduced by increasing the reactive diluent content. Rosu et al. [64] found a linear correlation between the reactive diluent content and the logarithm of viscosity, showing that the presence of reactive diluents accelerated the curing reaction and diminished the gel time. Dang et al. [62] proposed reinforcements for a comonomer vinyl ester (cVE) resin at different weight fractions of up to 2% via a direct polymerization process with a eutectic gallium–indium (EGaIn) alloy and graphene nanoplatelets, showing that sub-micron sized EGaIn (≤1 wt.%) could promote the curing reaction of cVE without changing the curing mechanism (Figure 4B).

#### 2.2.4. Polydicyclopentadiene (p-DCPD)

Dicyclopentadiene (DCPD) is a commercially available monomer that is derived from low-viscosity petrochemicals, making it easy to impregnate into fibers. Due to its impregnation characteristics, its market revenue reached approximately USD 0.86 billion in 2020 and is expected to grow at a CAGR of 5.7% between 2022 and 2030 [65]. Polydicyclopentadiene (PDCPD) is a highly crosslinked polymer formed by the ring-opening metathesis polymerization (ROMP) of its monomer precursor. Exothermic characteristics were observed during the polymerization process because of the relief of the ring strain energy initiated by the transition-metal/alkylidene complexes. Several studies investigated the effects of these catalysts.

**Figure 5:**
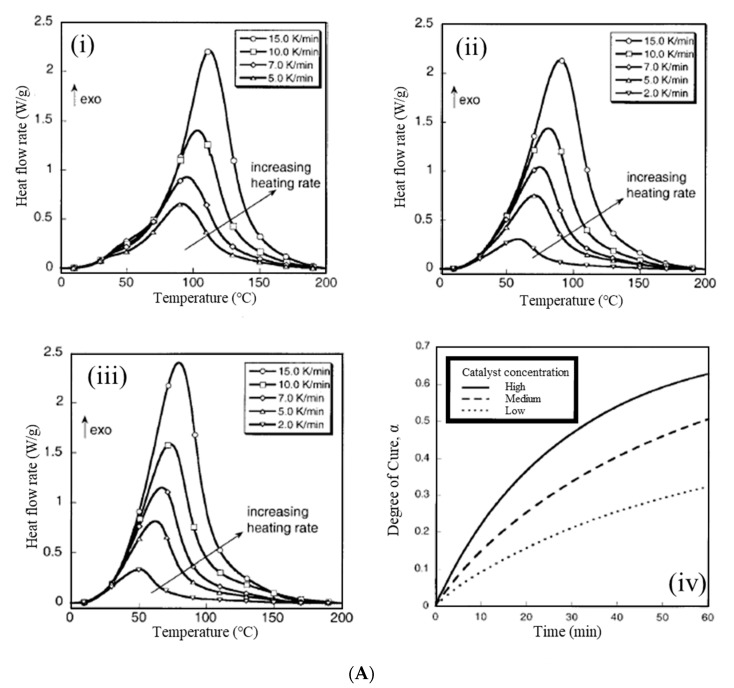

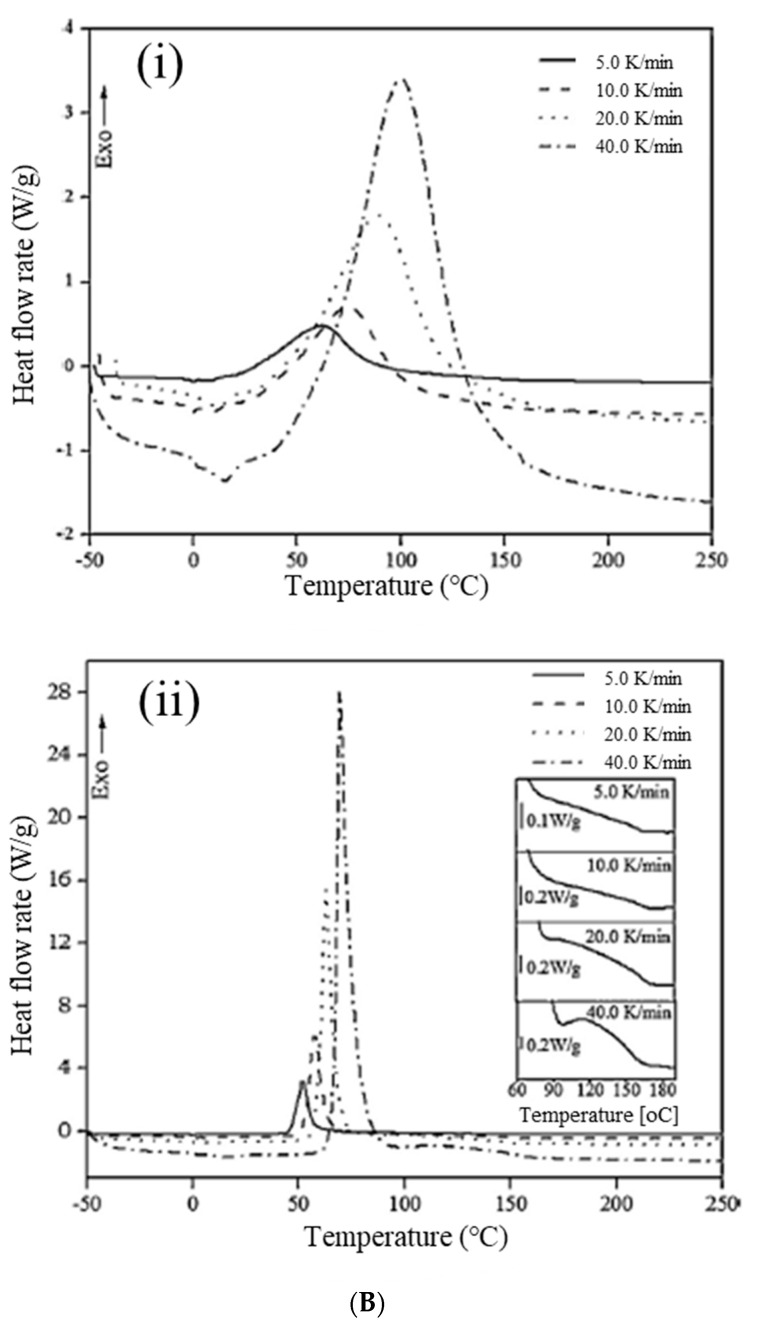
(**A**) The DSC curves for (**i**) low concentration (**ii**) medium-concentration, and (**iii**) high−concentration DCPD and Grubbs’ catalyst samples; (**iv**) predictions for isothermal curing at 30 °C based on the model−free iso−conversional method for low, medium, and high catalyst concentrations. Reproduced with permission [67]. Copyright 2002, John Wiley and Sons. (**B**) DSC scans at different heating rates for endo−DCPD with (**i**) 1st generation and (**ii**) 2nd generation Grubbs’ catalysts (inset shows the shoulder region). Reproduced with permission [68]. Copyright 2013, Elsevier.

Li et al. [66] conducted the ROMP of DCPD using the catalyst systems, WCl6–Et2AlCl and (WCl6–PhCOMe)–Et2AlCl, and their polystyrene-supported counterparts. The acetophenone-modified catalyst system exhibited better catalytic properties than the unmodified system. Moreover, as the polymer yield of ROMP increased, the mechanical properties of notched impact strength (NIS) and the tensile strength (TS) of the synthesized PDCPD increased. Kessler et al. [67] investigated the curing kinetics of PDCPD, prepared via ROMP, with three different concentrations of Grubbs’ catalyst using differential scanning calorimetry (Figure 5A). The catalyst concentration had a large effect on the curing kinetics, and the activation energy increased significantly at 30 °C. Yang and Lee [68] investigated the curing kinetics of endo-dicyclopentadiene (DCPD) with two types of Grubbs’ catalysts (1st and 2nd generation), using dynamic DSC at different heating rates (Figure 5).

Experimental DSC data obtained at different heating rates were used to evaluate the kinetic parameters of the model-free iso-conversional and model-fitting methods. In the single DSC exotherm of the 1st generation system (Figure 5(Ai)), the appearance of a shoulder above the single exotherm of the 2nd generation system (Figure 5(Aii)) suggests that reaction mechanisms other than ROMP, regarding the norbornene and cyclopentene units, may be involved in this catalyst system. The 2nd generation catalyst system showed a slower initiation rate but a faster polymerization rate compared with the 1st generation.

**Figure 6:**
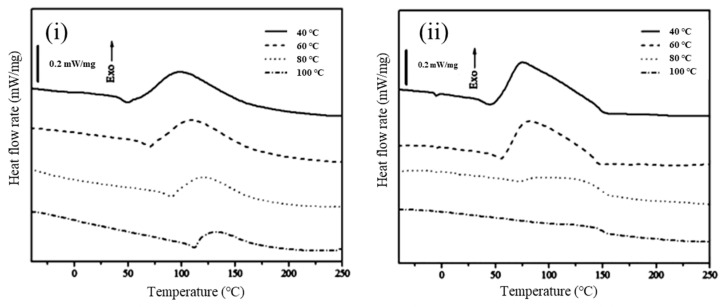

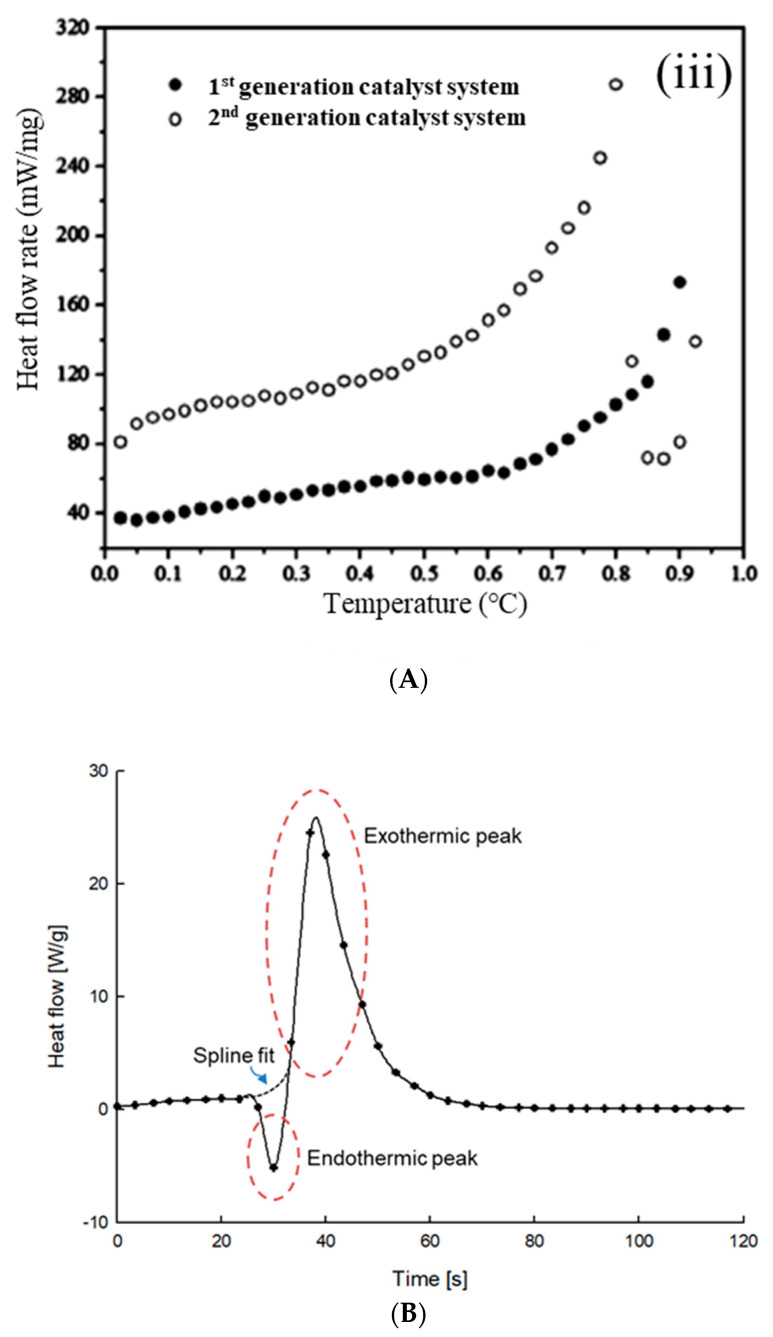

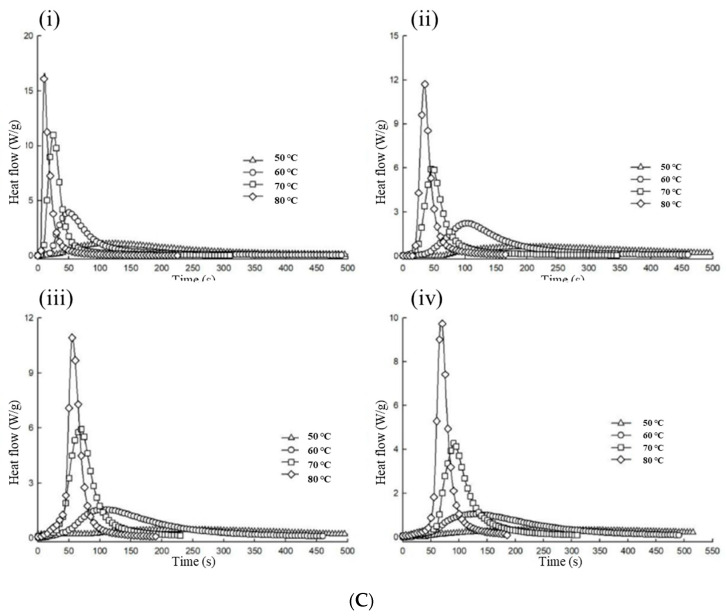
(**A**) Dynamic DSC scans following the isothermal cure of endo-DCPD with (**i**) the 1st generation and (**ii**) the 2nd generation Grubbs’ catalysts. (**iii**) Activation energy as a function of the fractional conversion of endo-DCPD with the 1st and the 2nd generation Grubbs’ catalysts. Reproduced with permission [69]. Copyright 2014, American Chemical Society. (**B**) Dynamic DSC curve with endo-DCPD with different amounts of decelerators. Reproduced with permission [70]. Copyright 2019, Elsevier. (**C**) Isothermal DSC profiles of endo-DCPD with different amounts of decelerators, as follows: (**i**) 0.5 wt. mass %, (**ii**) 1.0 wt. mass %, (**iii**) 1.5 wt. mass %, (**iv**) 2.0 wt. mass %. Reproduced with permission [70]. Copyright 2019, Elsevier.

Yang and Lee [69] also studied two Grubbs’ catalysts that exhibited apparent differences in the isothermal curing of endo-dicyclopentadiene (endo-DCPD) via ROMP, using the 1st and 2nd generation Grubbs’ catalysts as polymerization initiators. The 2nd generation catalyst was more efficient than the 1st generation catalyst in terms of catalytic activity, as evidenced by the reaction rates and fractional conversions (Figure 6A).

**Figure 7:**
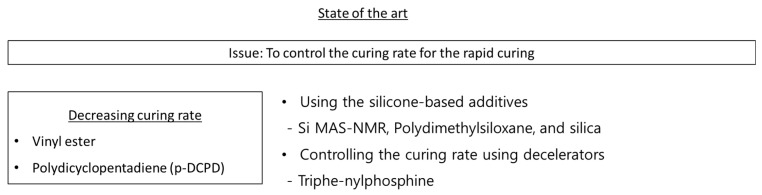
Recent state-of-the-art research on vinyl esters and DCPD [63,70,71].

Recent state-of-the-art research on vinyl esters and DCPD has focused on controlling the curing time; this is due to their extremely fast curing times, as shown in Figure 7. Yoo et al. [70] obtained the curing kinetics of endo-DCPD, using isothermal differential scanning calorimetry, by experimentally acquiring kinetic parameters in accordance with model-fitting approaches. Due to the rapid curing of DCPD, a decelerator was included in the manufacturing process. Therefore, the effect of the decelerator was investigated using the curing kinetics of endo-DCPD with different amounts of decelerator solutions, and it was found that the decelerator delayed the reaction and slowed the curing process (Figure 6B,C).

## 3. Rapidly Impregnating Thermoplastic Resins and Their Fiber-Reinforced Composites

In recent times, to a certain extent, thermoplastic composites (TPCs) have started to replace thermosetting composites and lightweight metal materials. Worldwide, the market value of TPCs increases every year, from 28 billion U.S. dollars in 2019 to an estimated 36 billion U.S. dollars by 2024; this is because they are very tough, the manufacturing process is faster, they are highly processable and recyclable, they are able to be welded, etc. [72].

Generally, the high melting viscosities of thermoplastic polymers require high processing temperatures and pressures to fully impregnate fibers and reduce defects in products [73]. Subsequently, in situ polymerization methods for fiber-reinforced TPCs have been developed using low-viscosity monomers or oligomeric precursors, such as caprolactam [74,75,76,77], laurolactam [78,79], methylmethacrylate (MMA) [80], and cyclic butylene terephthalate (CBT) [81,82], to fabricate fiber-reinforced polyamide 6 (PA6), polyamide 12 (PA12), polymethylmethacrylate (PMMA), and polybutylene terephthalate (PBT) composites, respectively. The global market size for PA 6 is estimated at USD 12.7 billion, and for PA 12, it is estimated at USD 19.43 billion [83]. For the PMMA, the global market size was expected to reach USD 8.33 billion by 2032 and USD 5382 million by 2029 [84]. These monomers (or oligomeric precursors) are polymerized via the addition of catalysts and activators. Table 1 lists several processing parameters and applications of commonly used monomers (or oligomeric precursors) with low viscosities that are suitable for LCM. In this section, we mainly introduce PA6, PA12, PMMA, and PBT thermoplastic composites, and we provide an overview of thermoplastic composites fabricated via in situ polymerization during LCM. Moreover, the effects of reactive processing parameters on the mechanical properties are discussed.

### 3.1. Polyamide 6 (PA6)

**Figure 8:**
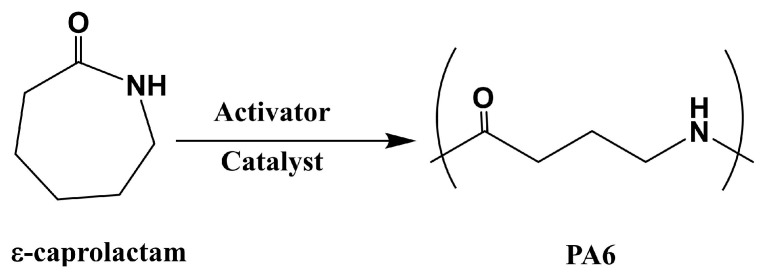
Schematic of the anionic ring open polymerization of PA6.

**Figure 9:**
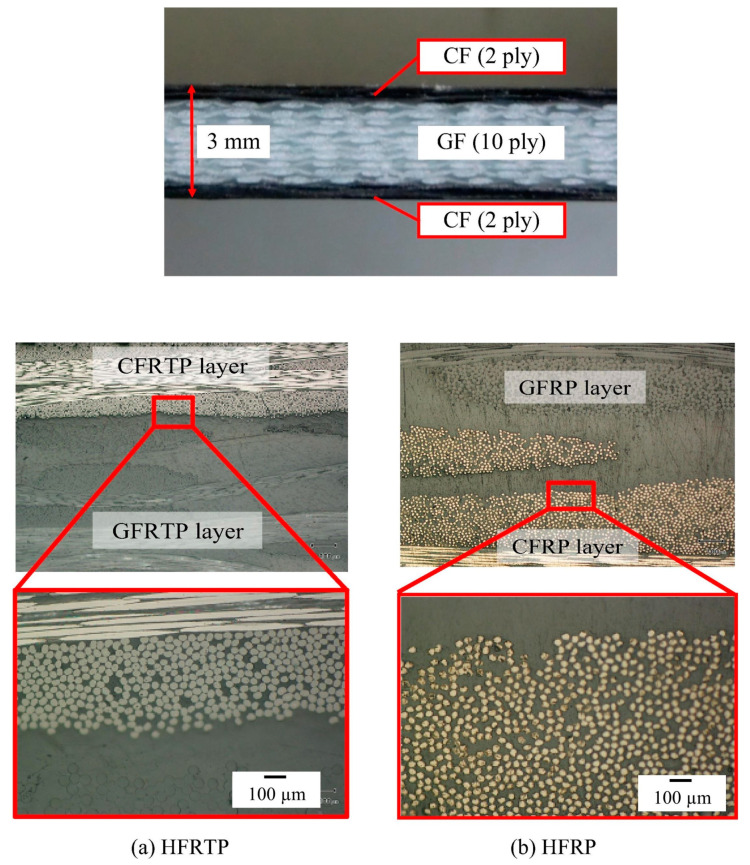
Cross sections of both plates: (**a**) hybrid, fiber-reinforced thermoplastic (HFRTP), (**b**) hybrid, fiber-reinforced plastic (HFRP). Reproduced with permission [75]. Copyright 2015, Elsevier.

PA6 was synthesized via the anionic ring-opening polymerization of ε-caprolactam, which is a crystalline cyclic amide with a melting temperature of 70 °C, and it is polymerized at 130–170 °C in the presence of a catalyst and activator [85] (Figure 8). PA6 based fiber-reinforced composites can be fabricated within 3–60 min, depending on the type and amount of the catalyst and activator used. Ahmadi et al. [91] suggested that the correct ratio of monomer, catalyst, and activator is a key component in anionic-caprolactam polymerization, and it provides the lowest monomer residue and best properties for the PA6 samples. In addition, polymerization time directly affects the production cycle and cost. Our previous research [76] focused on the effect of polymerization temperature on the degree of polymerization and polymerization time in order to produce perfect products with the shortest molding cycle time. The results showed that the polymerization and crystallization of PA6 occurred simultaneously during heating. As the heating rate increased, the crystallinity decreased, but the degree of polymerization increased. Furthermore, the viscosity of ε-caprolactam varied almost linearly with time in the early stages, whereas it increased exponentially from 20 s after the start of polymerization, indicating the presence of the injection molding cycle time. Ben et al. fabricated glass and carbon fiber hybrid PA6 composites (Figure 9) with A (caprolactam and activator) and B (caprolactam and catalyst) mixtures via vacuum-assisted resin transfer molding (VaRTM) in order to evaluate their mechanical properties when applied to automobile structures [75]. The results showed that the bending, tensile, and compressive strengths of the hybrid-fiber-reinforced PA6 were 594, 315, and 297 MPa, respectively, which were comparable to those of the hybrid, fiber-reinforced fast-curing epoxy (597, 327, and 318 MPa, respectively). However, the flammability of polyamides, which is a key issue in the automobile industry, limits their widespread application. The main challenges include the inhibition of in situ polymerization in the presence of flame retardants and the insolubility of flame retardants due to the filtration of reinforcements such as titanium dioxide, multiwalled carbon nanotubes, phosphorus compounds, etc. [92,93].

In addition, recycling is also an issue for PA6. As a non-degradable plastic, PA6 is extremely challenging to recycle, and it cannot be recycled using traditional methods. Recently, Wursthorn et al. [94] developed lanthanide trisamido catalysts, with which, PA6 can be depolymerized to ε-caprolactam with a high selectivity (more than 95%) and yield (more than 90%), and no solvents or toxic chemicals are used in the whole process. The generated ε-caprolactam can be used as monomers to obtain new PA6, thus, it is feasible to employ this method to recycle PA6 products.

### 3.2. Polyamide 12 (PA12)

**Figure 10:**
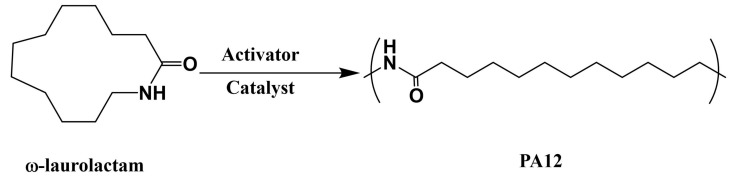
Schematic of the anionic ring open polymerization of PA12.

PA12 is called nylon 12, and it is synthesized via the anionic ring-opening polymerization of ω-laurolactam, as shown in Figure 10. ω-laurolactam has a low initial viscosity above its melting point at 153 °C, facilitating the easy and complete impregnation of fibers in the mold. Similar to PA6, PA12-based fiber-reinforced composites can be fabricated using LCM processes, such as thermoplastic resin transfer molding (T-RTM). The desired injection temperature was found to be 170–205 °C, and polymerization started at 180–250 °C, after introducing the catalyst and initiator. Mairtin et al. [79] developed carbon-fiber-reinforced PA12 composites, with a 60% carbon fiber volume fraction, which exhibited high tensile strength (788.3 MPa) and high compression strength (365.7 MPa). It is also reported that the polymerization time is related to polymerization temperature, that is, it takes 8.5 min and 20 min at molding temperatures of 240 °C and 200 °C, respectively.

**Figure 11:**
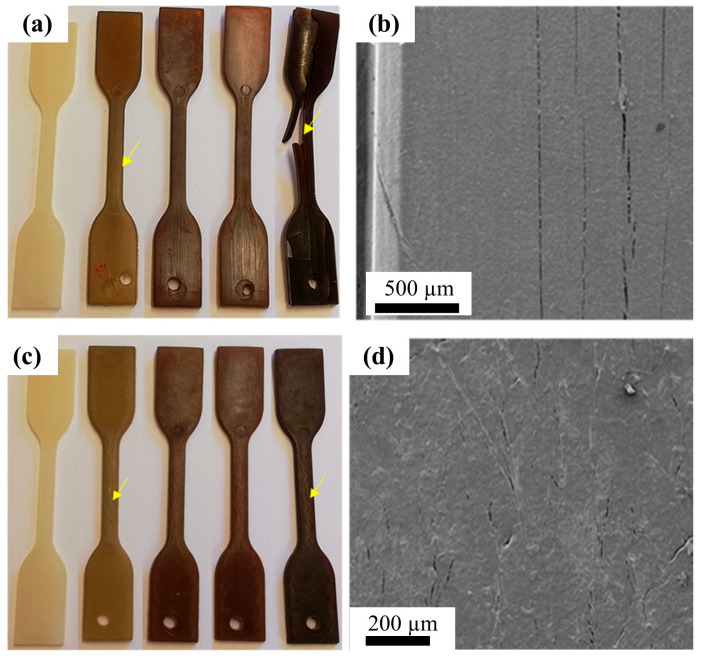
Images of (**a**) PA12 and (**c**) glass-fiber-reinforced PA12 composites (from left to right, samples aged for 0, 150, 300, 500, and 700 h). SEM images of the surface of the (**b**) 150 h-aged PA12 sample and (**d**) 150 h-aged glass-fiber-reinforced PA. Reproduced with permission [95]. Copyright 2022, Springer Nature.

As listed in Table 1, PA12 is commonly used in fuel filter housing and fuel pipe connectors, which are close to the engine and exposed to fuel and high service temperatures. Therefore, fuel uptake and aging behavior are important factors. Wei et al. [95] found that pure PA12 showed fast and remarkably high fuel uptake when exposed to a mixture of ethanol and gasoline at 120 °C; however, a lower uptake was observed for glass-fiber-reinforced PA12 composites. As shown in Figure 11, the PA12 and glass-fiber-reinforced PA12 composites gradually changed color from white to yellow as the exposure time increased; this is due to the oxidation of PA12, and the cracks in PA12 were larger than those in glass-fiber-reinforced PA12, indicating a suppression effect of glass-fiber on fuel uptake.

In addition, PA12 can be recycled and used in automobiles. It has been reported that an automobile fuel-line clip, produced with recycled PA12 through a selective laser sintering method, provides an 8% reduction in life-cycle global warming potential and life-cycle primary energy demand compared with conventional PA66 [96], thus improving sustainability properties.

### 3.3. Polymethyl Methacrylate (PMMA) (Elium^®^)

**Figure 12:**
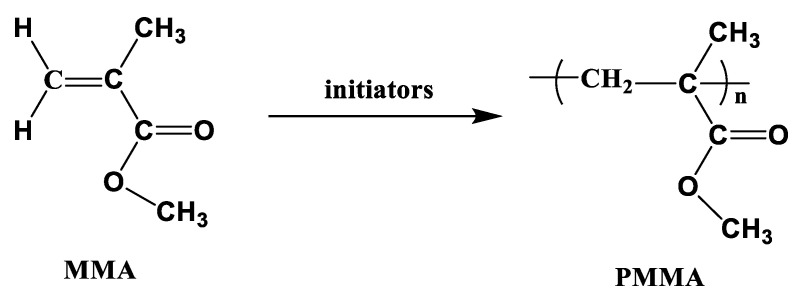
Schematic of the vinyl polymerization of PMMA.

PMMA is extensively used in the automobile industry to produce various parts and components of vehicles, such as external, rear, and indicator light covers; decorative trims; ambient lighting; door entry strips; and automobile glazing. This is due to its light weight, high scratch resistance, and low stress birefringence. As shown in Figure 12, PMMA was synthesized via the free radical vinyl polymerization of methylmethacrylate (MMA) in the presence of peroxide initiators. The melting temperature of MMA is −48 °C, and it is polymerized at relatively low temperatures (120–160 °C); however, the boiling temperature of MMA is 100 °C, which means that it boils easily and can cause voids in the final products. Moreover, a long cycle time (>900 min) was required to fully polymerize MMA below its boiling temperature.

**Figure 13:**
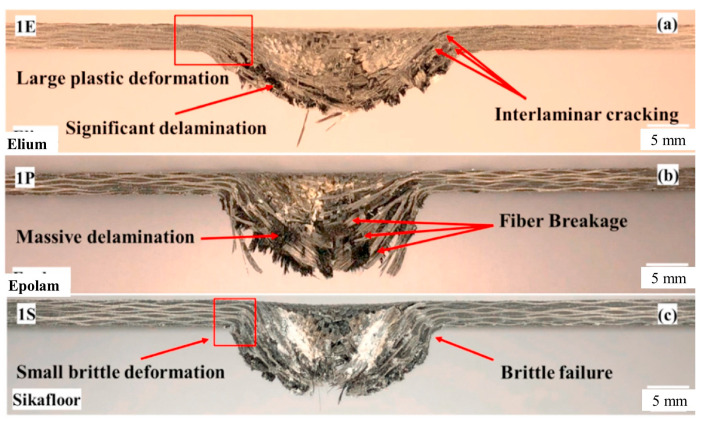
A comparison of cross-sectional observations of (**a**) the carbon-fiber-reinforced Elium^®^ and (**b**,**c**) thermosetting epoxy (Epolam, Sikafloor) laminates at 20 J. Reproduced with permission [97]. Copyright 2021, Elsevier.

Recently, a novel liquid-reactive MMA has been developed by Arkema, named Elium^®^. It has low viscosity and low processing temperature (room temperature), and it is used in conjunction with a dibenzoyl peroxide initiator [88]. Elium^®^ can also be used to impregnate fibers via the LCM process, which is the same method used as traditional MMA. Several studies have been conducted to evaluate its mechanical properties. Kazemi et al. [97] studied the dynamic response of carbon-fiber-reinforced Elium^®^ and carbon fiber-reinforced epoxies (Epolam, Sikafloor) using low-velocity impact tests. This study demonstrated the higher plasticity of Elium^®^-based composites compared with epoxy-based composites, resulting in less structural loss and less absorbed energy, as shown in Figure 13. In addition, many studies have reported on the good mechanical properties of Elium^®^-based fiber-reinforced composites, such as good toughness, flexural and tensile strength, welding performance, etc. [98,99,100]. However, Elium^®^ has a much higher shrinkage rate than that of common PMMA due to its fast polymerization, which is a problem to be solved in future studies [101].

In addition, some recycling technologies for Elium^®^, such as the mechanical recycling method and chemical recycling method, have already been developed to obtain recycled materials or they have been recovered as monomers [102]. Generally, these recycled materials are reused with virgin materials to enhance mechanical properties, however, recovered monomers could be polymerized to obtain new products. Though a few studies on the characterization and analysis of recycled products have been investigated, more intensive work should be needed to evaluate the life cycle of these recycled products.

### 3.4. Polybutyleneteraphthalate (PBT)

**Figure 14:**
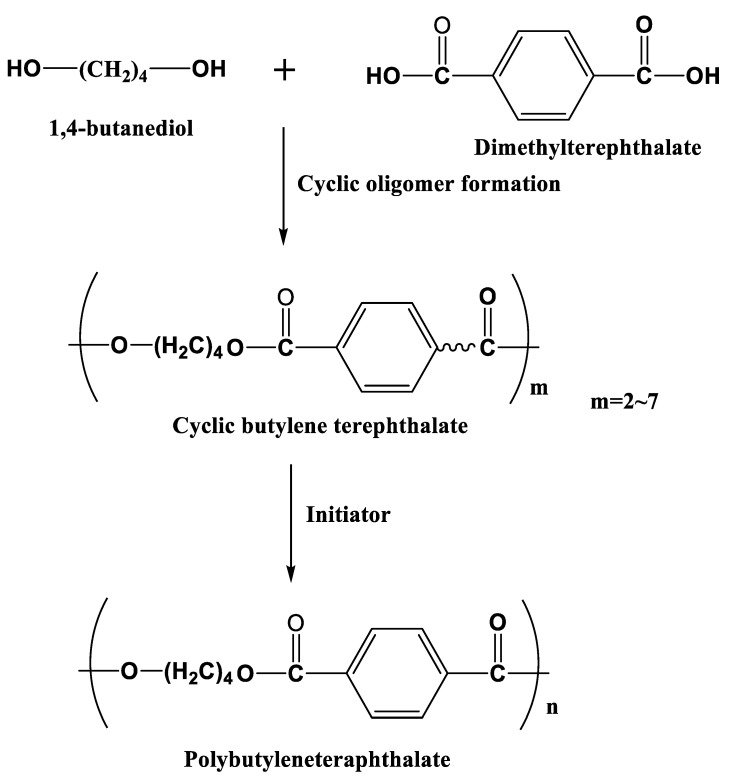
Schematic of the anionic ring open polymerization of PBT.

PBT is widely used in the automobile industry owing to its high stiffness and strength. Indeed, 1,4 butanediol and dimethyltetrephthalate are used as monomers to produce macrocyclic oligomers of CBT with two to seven repeat units [103], and this is followed by the polymerization of semicrystalline PBT in the presence of an initiator (Figure 14). The initial viscosity of CBT is 20 mPa⸱s at 190 °C, which is suitable for the LCM processing of, for instance, RTM [104]. It is reported that PBT polymerized from CBT via RTM is more brittle than that of conventional PBT due to the high crystallinity of the polymerized PBT [105]. Its toughness could be improved with the addition of nanoparticles [106,107], fibers [108], etc. Baets et al. found that the addition of 0.05–0.1 wt.% of multi-walled carbon nanotubes (MWCNTs) could increase the toughness, stiffness, and strength of PBT composites [109]. They also prepared polycaprolactone-blended CBT/glass-fiber composites to improve the toughness of the composites [109]. Yang et al. [110] found that woven carbon fabric and glass fabric hybrid PBT composites, which are fabricated via a vacuum assisted prepreg process, have a higher impact resistance than that of PBT/carbon fiber (CF) composites, although the presence of fibers may reduce the conversion of CBT. Furthermore, non-isothermal production processes, solvent blending, the addition of plasticizers, and chemical modification can enhance the toughness of CBT composites [81,111,112].

In addition, PBT can be recycled via depolymerization into CBT or monomers (1,4-butanediol and dimethyltetrephthalate) which exhibit properties comparable to those of baseline materials. Cao et al. [113] prepared super tough PBT/MWCNT/epoxidized elastomer composites with excellent mechanical properties for a wide range of PBT applications in the automobile industry.

Despite significant progress, several challenges persist in terms of the rapid impregnation of thermoplastic resins to produce fiber-reinforced composites in the automobile industry. These challenges include achieving uniform resin distribution, controlling fiber wetting, minimizing void content, and maintaining mechanical properties. Recent advancements have focused on addressing these challenges through innovative approaches [41,114]. Furthermore, thermoplastic-based automobile parts are also required to increase automotive plastic reuse, recycling, and recovery, in order to reduce overall automotive plastic waste generation for environmental sustainability. Many studies have reported that plastic parts could be reused or recovered from end-of-life vehicles, and some parts could be recycled via high-vacuum extraction, melt filtration, introducing additives, and so on. However, it is challenging to recycle fiber-reinforced plastic composites or multi-component-blended composites. Recently, a physicochemical recycling method has been developed to recover matrices and fibers which preserve the fibers’ lengths [115]. Furthermore, more intensive work on the environmental impacts and life cycle assessments of these recycled products should be investigated [116,117].

## 4. Current Research Gaps and Future Research Outlook

The current research concerning rapidly impregnating resins and the production of fiber-reinforced composites in the automobile industry has identified several research gaps. Addressing these gaps and focusing on future research can lead to advancements and improvements in this field. Here are some of the current research gaps and potential future research directions [118,119,120].

Enhanced impregnation efficiency: Achieving the uniform impregnation of reinforced fibers with resin is critical for high-quality composites. Research has focused on exploring different impregnation techniques and parameters to minimize voids, ensure uniformity, and enhance interfacial adhesion. Although progress has been made in terms of impregnation techniques, there is a need to further enhance the impregnation efficiency of rapidly impregnating resins, which would enhance the amount of pore space penetrated by the resin [121,122,123,124]. Future research should focus on improving resin flow and wetting behavior to achieve the better impregnation of reinforcing fibers. This includes studying the effects of resin viscosity, fiber architecture, and processing parameters on impregnation efficiency.

Optimization of curing processes: A rapid curing process is crucial for the efficient production of fiber-reinforced composites [10,125]. Future research should aim to optimize curing processes by investigating advanced heating methods, optimizing curing temperatures and times, and exploring the use of catalysts or additives to accelerate the curing reaction. Such research will help reduce cycle times and improve the overall productivity of composite manufacturing. 

Characterization and optimization of mechanical properties: Understanding and tailoring the mechanical properties of rapidly impregnating resins for specific automotive applications is essential. Future research should focus on exploring the development of new resin formulations that are specifically designed for rapid impregnation; this will involve modifying the viscosity, curing kinetics, or surface tension of the resin to improve its flowability and fiber wetting characteristics. In addition, the resin composition, placement of fiber reinforcement, and processing conditions should be optimized to achieve the desired mechanical properties. This can be achieved through a combination of experimental testing, numerical modeling, and material characterization techniques [126,127,128].

Durability and long-term performance: Regarding environmental issues, sustainable fibers are of great interest to the automotive industry. However, automotive parts are usually exposed to environmental factors, such as UV radiation, temperature, humidity, and chemical exposure, resulting in poor interfacial properties, water absorption, swelling, etc. [129,130]. Therefore, future research should focus on developing bio-based materials and enhancing the resistance of the composites, as well as their degradation mechanisms [131,132]. 

Environmental sustainability and recyclability: Given the increasing environmental concerns, future research should focus on developing sustainable and recyclable rapidly impregnating resins [133]. This includes exploring the use of bio-based or recycled materials as resin matrices, investigating recycling techniques for end-of-life composites, and assessing the environmental impact of these materials throughout their lifecycle.

By addressing these research gaps in future research, the automobile industry can benefit from improved rapidly impregnating resins that offer enhanced impregnation efficiency, optimized curing processes and mechanical properties, and improved durability and sustainability.

## 5. Conclusions and Outlook

The impregnation performance of thermoset resin and thermoplastic resin is crucial for manufacturing composite materials like glass-fiber or carbon-fiber-reinforced polymers. The resins act as a matrix that bind the fibers together, providing strength, stiffness, and durability to the composites. They enable the production of lightweight, yet high-performance, materials that are widely used in aerospace, automotive, and construction industries. One key aspect of impregnating resins is their ability to improve the structural integrity of composite materials. By impregnating fibers or porous structures, the resins enhance the strength, stiffness, and impact resistance of the composite. This is particularly important in industries where lightweight and high-performance materials are sought after, such as in the aerospace and automotive sectors. Therefore, impregnating resins are of significant importance in the polymer resin market due to their ability to enhance the mechanical properties, durability, and protection of materials; this highlights their value and the demand for such specialized resins.

In this paper, rapidly impregnating resins for fiber-reinforced composites are discussed as alternatives to high-performance metal components. An overview of suitable rapidly impregnating resins with low viscosities are introduced, and the differences between thermoset and thermoplastic composites are identified. 

Thermoset resins, such as epoxy, polyester, vinyl ester, and DCPD, have excellent dimensional and chemical stabilities and high impact strengths. The epoxy-dicyandiamide system, as a representative commercial epoxy resin, has many strategies which have been implemented to develop low-viscosity, fast-curing epoxy resins, such as the addition of a GDE series and synthesized epoxies. The reaction time decreased with the addition of various particles. The reinforcement of low-viscosity unsaturated polyester resins has also been introduced. Low-viscosity polyester resins can be obtained via particle synthesis. The curing time can be reduced by using various solvents and applying microwaves. Low viscosity, coupled with a rapid curing rate at room temperature, and the relatively low cost of vinyl ester resins, can be obtained using dispersants and various acids to reduce the surface-active properties. 

Regarding the thermosetting resins, PA-6, PA-12, PMMA (Elium^®^), and PBT were introduced, which have high melting viscosities, and they require a high processing temperature and pressure to fully impregnate the fibers and reduce defects in the products. Therefore, in situ polymerization methodologies for fiber-reinforced thermoplastic composites with low viscosities have been developed, and they are suitable for liquid molding processes. 

Overall, extensive studies have been conducted on the characterization, analysis, and simulation of rapidly impregnating, resin-based, fiber-reinforced composites. However, the large-scale production of such composites has been rare. Therefore, future research should focus on the large-scale production of composites for the automobile industry, a reduction in their manufacturing time, and an improvement in their performance. In addition, as environmental regulations become stricter, the requirements of automobile materials are also becoming stricter. Some heavy metals and organic substances are banned or restricted to use in automobiles, and automobile parts which cannot be further divided should be merged with homogeneous resins so that they can be recycled more efficiently. Therefore, alternative materials should satisfy the harmlessness to the human body and the environment, and the material itself also needs to satisfy certain performance criteria so that they are comparable to fiber-reinforced or polymer-blended composites. As technologies and industries continue to advance, the importance of rapidly impregnating resins is expected to grow, driven by the need for improved performance, longevity, and reliability of materials and products.

## Figures and Tables

**Table 1:** Processing temperatures and processing times of various monomers.

	Monomer	Processing Temp. (°C)	Processing Time (min)	Application	Ref.
Thermoset	Epoxy (glycol diglycidyl ether (GDE) series diglycidyl ether of bisphenol F, diethyl toluene diamine, and 2-ethyl-4-methylimidazole)	120	180	Aerospace, marine, automobile, construction.	[25,26,27]
	Polyester (polyethylene terephthalate (PET))	40–80	120	Construction, marine, chemical.	[57]
	Vinyl Ester (vinyl acetate, vinyl propionate, and vinyl laurate)	50–200	<240	Coatings, printed circuit boards, building materials, automotive parts, and fiber-reinforced composites.	[36,37,63]
	p-DCPD (Dicyclopentadiene monomer)	50–90	2	Sporting goods, automotive industries, as well as military and aerospace applications.	[70]
Thermoplastic	PA-6 (ε-caprolactam)	130–170	3–60	Wipers, gears, bearings, and weatherproof coatings.	[85]
	PA-12 (ω-laurolactam)	180–240	with cooling process	Fuel pipes, fuel filters, high pressure oil pipes, gears, brake hoses.	[86,87]
	PMMA (Elium^®^) (vinyl polymerization of methylmethacrylate (MMA))	80–160 (room temp.~)	low emp.: >900 high temp.: Boiling of monomer	Decorative trims, interior lighting, door entry strips.	[85,88]
	PBT (1,4 butanediol and dimethyltetrephthalate)	180–250	<30	Door handles, bumpers.	[72,89,90]

## Data Availability

The data presented in this study are available on request from the corresponding author.
